# Development of Hybrid Braided Composite Rods for Reinforcement and Health Monitoring of Structures

**DOI:** 10.1155/2014/170187

**Published:** 2014-01-20

**Authors:** Sohel Rana, Emilija Zdraveva, Cristiana Pereira, Raul Fangueiro, A. Gomes Correia

**Affiliations:** ^1^Fibrous Materials Research Group (FMRG), School of Engineering, University of Minho, Azurem Campus, 4800-058 Guimaraes, Portugal; ^2^Department of Civil Engineering, University of Minho, Azurem Campus, 4800-058 Guimaraes, Portugal

## Abstract

In the present study, core-reinforced braided composite rods (BCRs) were developed and characterized for strain sensing capability. A mixture of carbon and glass fibre was used in the core, which was surrounded by a braided cover of polyester fibres. Three compositions of core with different carbon fibre/glass fibre weight ratios (23/77, 47/53, and 100/0) were studied to find out the optimum composition for both strain sensitivity and mechanical performance. The influence of carbon fibre positioning in BCR cross-section on the strain sensing behaviour was also investigated. Strain sensing property of BCRs was characterized by measuring the change in electrical resistance with flexural strain. It was observed that BCRs exhibited increase (positive response) or decrease (negative response) in electrical resistance depending on carbon fibre positioning. The BCR with lowest amount of carbon fibre was found to give the best strain sensitivity as well as the highest tensile strength and breaking extension. The developed BCRs showed reversible strain sensing behaviour under cyclic flexural loading with a maximum gauge factor of 23.4 at very low strain level (0.55%). Concrete beams reinforced with the optimum BCR (23/77) also exhibited strain sensing under cyclic flexural strain, although the piezoresistive behaviour in this case was irreversible.

## 1. Introduction

Light weight combined with very good mechanical properties has opened up the possibility of using fibre reinforced plastics (FRPs) in several high end applications including reinforcement of concrete structures. The main disadvantage of steel bars commonly used for concrete reinforcement is the corrosion problem. The use of FRPs in civil structures as replacement of steel is gaining popularity due to their light weight and very good corrosion resistance. Moreover, FRPs offer huge flexibility in tailoring their properties by selecting different fibre/matrix systems and composite structures. Therefore, it is possible to introduce different functionalities into FRPs so that they can serve several purposes at the same time. Health monitoring of civil structures is very much essential in order to avoid accidents from sudden fractures. The approach of inserting various types of sensor (strain gauges, piezoelectrics [1, 2], fibre optics [3, 4], etc.) into the structures for health monitoring is usually complex and expensive and requires highly skilled personnel for their application and use. These problems can be overcome by turning concrete structures intelligent, that is capable of sensing their own deformation and damage. FRPs are the ideal reinforcements for designing such concrete structures due to the possibility of imparting multifunctionality to FRPs by tailoring their composition or structure. Efforts have been directed towards designing FRPs with self-sensing capability by introducing into their structure a conductive component such as carbon fibres, which can change their electrical resistance with deformation and damage. Use of hybrid composites containing mixture of conductive reinforcements with other reinforcements such as glass and aramid proved helpful to introduce pseudoductility in order to detect damage well before the structural collapse [5–7]. Several hybrid composite systems with different structures and compositions of glass and carbon fibres or powder have been investigated in order to develop either continuous monitoring system [7–9] or discontinuous warning system for structural health monitoring [10, 11]. Some researchers succeeded to develop hybrid carbon/glass fibre composites which are able to provide alarm signal well before the composite collapse [11]. However, a very small change in resistance at low strain level makes these hybrid composites unsuitable for designing continuous monitoring system [9]. Combination of glass fibre with carbon particle instead of continuous carbon fibre was found to improve the strain sensitivity at low strain level [12, 13]. Recently, attempts have also been made to develop hybrid composites with good sensitivity using carbon nanotubes (CNTs) [14–20]. However, dispersion of CNTs within the composite matrix is a critical issue, since improper dispersion can adversely affect the mechanical properties of composites [21]. Design of continuous monitoring system can also be based on the measurement of residual resistance caused due to deformation. However, it was observed that good sensitivity at low strain in terms of residual resistance is obtained only at the prestressed conditions [9]. Although prestressing of composites is a suitable condition for civil engineering structures, it may present problems in other applications.

Braiding process is gaining a lot of importance in recent times for the manufacturing of composites with complex structures due to its simplicity and low cost [22]. Core-reinforced braided composite rods (BCRs), which are comprised of unidirectional core fibres surrounded by a braided cover, have already been proposed for applications like concrete rebars and medical implants due to their very good mechanical performance [23, 24]. A simple and cost-effective process for continuous single step production of core-reinforced braided rods has been patented by the authors [25]. Superior adhesion of these braided rods with concrete as well as their applicability for concrete internal reinforcement as replacement of steel rebars has also been demonstrated by them [26, 27]. It was observed that BCRs containing carbon fibre core or hybrid cores containing mixture of carbon with glass or high tenacity polyethylene showed higher tensile strength and lower elastic modulus as compared to steel rebars [28]. Attempts are also being made currently to improve the modulus and ductility of BCR using certain proportion of steel fibres in the composition [29]. Potential of core-reinforced braided rods for structural health monitoring was also introduced in our previous studies [30, 31]. In these studies, BCRs with three core compositions (glass/carbon ratio of 23/77, 53/47, and 0/100) were characterized for piezoresistivity under cyclic flexural strain and the composition with lowest amount of carbon fibres (23/77) resulted in better strain sensing behaviour with a gauge factor as high as 23.4 [31]. The present paper reports the further continuation of this research work which also investigated the influence of core fibre positioning in the cross-section of composite rods on the strain sensing behaviour. Moreover, since reinforcement is also one of the primary tasks of BCRs, the effect of their composition on the mechanical properties has also been reported and discussed. Also, the probable mechanism of the piezoresistivity of BCR under flexural deformation has been discussed. In addition to that, in order to investigate the sensing ability of developed BCRs within concrete structures, mortar beams reinforced with BCRs having optimum composition were fabricated and characterized for piezoresistive behaviour under cyclic flexural strain.

## 2. Experimental

### 2.1. Materials and Methods

**Figure 1:**
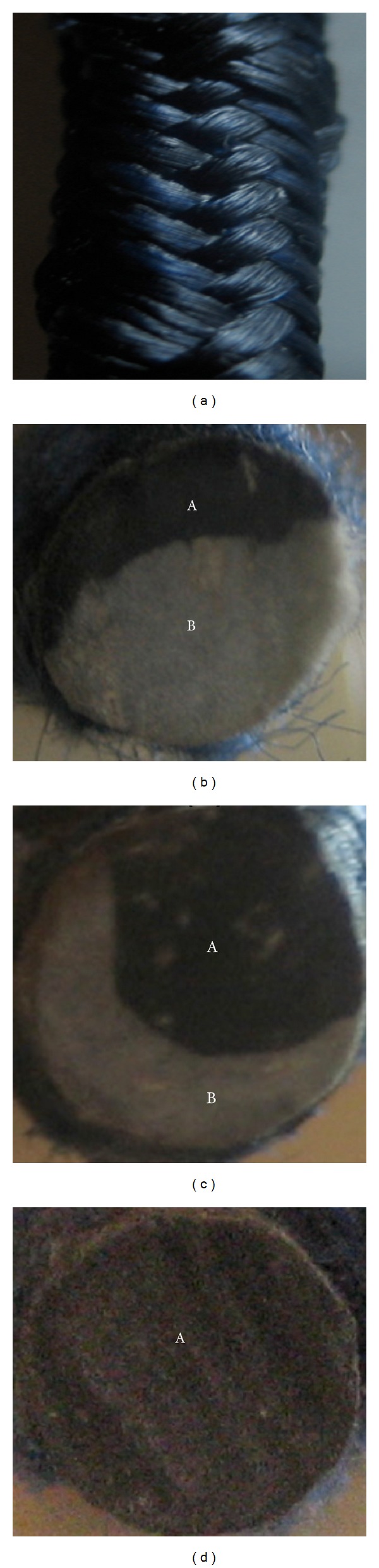
Surface texture of BCR (a) and distribution of carbon fibre (A) and glass fibre (B) within BCR1 (b), BCR2 (c), and BCR3 (d).

BCRs were produced using polyester fibres for braided structure and a combination of glass fibre and carbon fibre as the core reinforcement. Braiding of polyester fibres and impregnation of core fibres with polyester resin/hardener mixture were done simultaneously in a single process using a vertical braiding machine with an incorporated impregnation system [25]. The take-up speed was kept at 0.01 m/s, which led to a breading angle of 23-24°. The composite rods were then cured at environmental temperature and moisture conditions (20 ± 2°C and 50 ± 5%). The properties of E-glass and carbon fibres used for core reinforcement are provided in Table 1 and the composition of different composites is listed in Table 2. The surface texture and cross-sections of the braded rods have been illustrated in Figure 1. The ribbed surface texture leads to superior adhesion of these composite rods with concrete [26]. It can be noticed that in the hybrid rods, the carbon fibres are present in one side of the cross-section and the other side is comprised of glass fibres. The placement of the fibres within the cross-section of the braided rods was controlled while feeding them in the braiding machine by passing the fibres through holes of metal plates placed before and after the resin bath [25]. Carbon and glass yarns were distributed in these holes according to their position in the cross-section of BCRs. However, the core fibres were not put under any tension during feeding. As a result, they lost their straightness to some extent due to the braiding of polyester fibres around them and took a misaligned arrangement decided by the braiding process parameters such as braiding angle, take-up speed, and pretensioning [27]. The extent of this core fibre misalignment can be estimated from the strain of toe region (where mainly orientation of core fibres occurs due to tension leading to very small increase in load with deformation) present in the tensile curves of BCRs tested without pretension.

**Figure 2:**
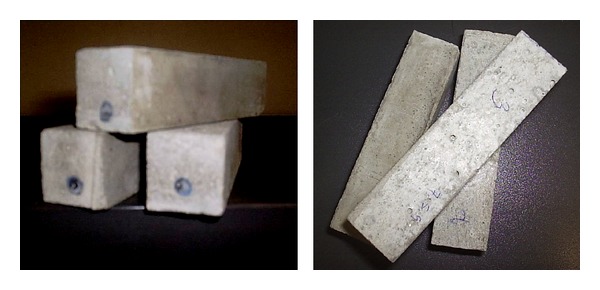
Mortar beams reinforced with BCRs.

BCR reinforced mortar samples were prepared by incorporating the BCRs within mortar paste (mixture of cement, sand, and water) according to 196_1_NPEN_1996 standard. A water/cement ratio of 0.5 was used and mixing of cement/water/sand was performed in a standard mixer. Demolding was done after 24 hours and demoulded samples were kept in a condition room with maintained temperature of 22°C and relative humidity of 65% for 14 days for setting. Samples for piezoresistive testing had the dimension of 2.5 cm × 2.5 cm × 10.5 cm (shown in Figure 2) with the braided rods located at ~5.5 mm from the bottom surface. Based on the results of mechanical properties and piezoresistive behaviour of BCR, mortar samples for piezoresistive characterization were prepared only incorporating BCR1 type of braided rod.

### 2.2. Characterization

**Figure 3:**
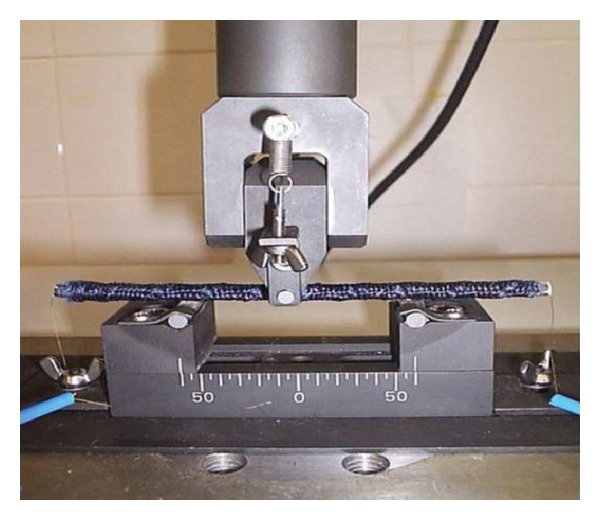
Measurement setup for piezoresistive characterization of BCR.

The strain sensing behaviour of BCRs was characterized by measuring the change in electrical resistance between the sample ends using two-terminal dc method [28] under cyclic 3-point flexural loading. Hybrid composites studied previously [32] showed poor strain sensing behaviour in flexural loading as compared to tensile or compression mode due to the nullifying effect of resistance change in the tension and compression side. Therefore, attempts have also been made by some researchers to measure the resistance change in tension and compression side separately [33]. However, the two-terminal resistance measurement between the sample ends, as employed in this study, is more simple and suitable for practical applications. Cyclic tests were performed at low strain range (up to 0.55%) in order to investigate the performance of the BCRs in sensing very low deformation in continuous manner. The experimental setup for the characterization of piezoresistive behaviour is shown in Figure 3 and the testing parameters are provided in Table 3. Electrical resistance of the samples was continuously measured during the flexural test by making electrical connections between the two probes of a digital multimeter (Agilent 84401A) and sample ends through gold wires fixed to the samples using silver paste. Since under flexural loading one side of the specimen is subjected to tensile stresses and the other side to compressive stresses, each BCR was tested two times keeping the carbon fibre part in tension and compression side, respectively. The objective here was to investigate the effect of carbon fibre positioning on the piezoresistive behaviour.

The strain sensing capability of the composites was evaluated in terms of gauge factor (GF), which is defined as follows:

(1) $$\begin{array}{c} \text{GF}=\frac{\Delta R/R}{\varepsilon }, \end{array}$$

where Δ*R* is the change in electrical resistance, *R* is the initial resistance, Δ*R*/*R* is the fractional change in resistance, and *ε* is the flexural strain at the outer surface of specimen at midspan, which was calculated from maximum deflection in the center of the rod (*D*), diameter (*d*), and support span (*L*) using the following formulae:

(2) $$\begin{array}{c} \varepsilon =\frac{6Dd}{L^{2}}. \end{array}$$

The fractional resistance change and gauge factor can be positive or negative depending on the type of response (positive response in case of resistance increases with deformation or negative response in case of resistance decreases with deformation). However, to avoid confusion only the magnitude of fractional resistance change and gauge factor has been reported mentioning the type of response separately. The braided composites were characterized for tensile properties in a Universal Testing Machine (Hounsfield H100 KS) according to ASTM D3916-94 using a crosshead speed of 5 mm/min.

**Figure 4:**
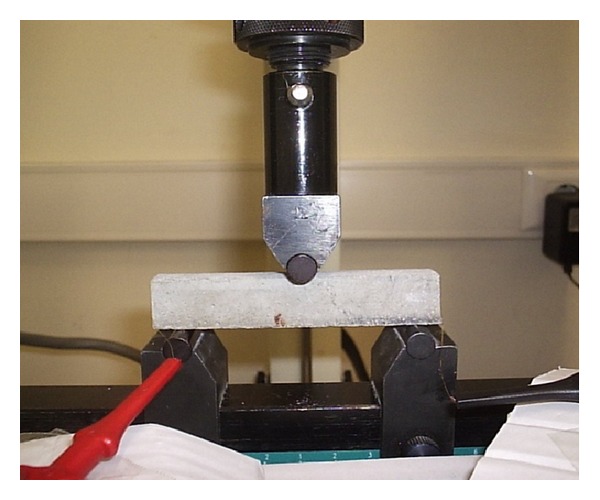
Measurement setup for piezoresistive characterization of BCR reinforced beams.

Piezoresistive behaviour of BCR reinforced mortar samples was characterized using a similar setup (Figure 4) as used for BCR characterization. The parameters for piezoresistive characterization of mortar samples are provided in Table 4. The mortar samples were characterized keeping the BCR in the tension side of the specimens. 3 samples from each category were tested to characterize the piezoresistive behaviour of BCR and reinforced mortar beams and the average value has been reported. In case of tensile testing of BCR, 5 samples were tested from each category.

## 3. Results and Discussion

### 3.1. Piezoresistive Behaviour

**Figure 5:**
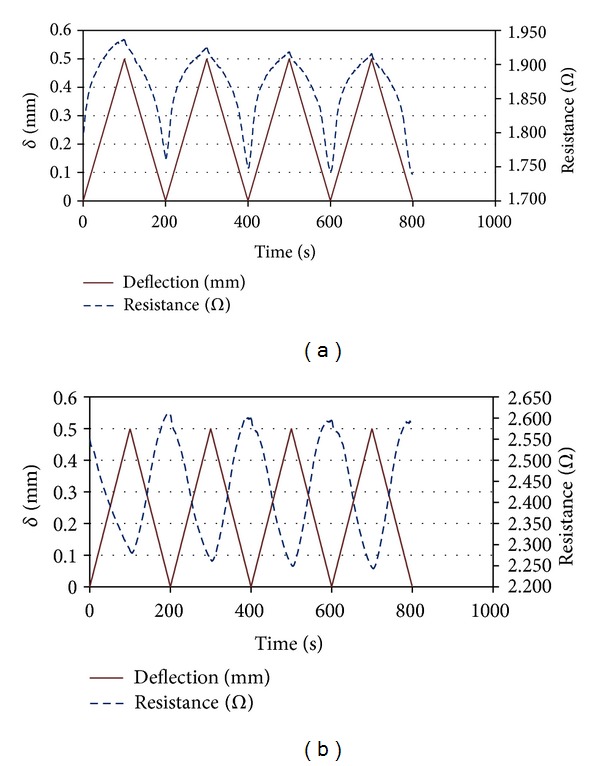
Piezoresistive behaviour of BCR1 in cyclic flexural loading: (a) positive response and (b) negative response.

The developed BCRs showed two types of piezoresistive behaviour under flexural loading depending on the position of carbon fibres in the cross-section of braided composites. If the carbon fibres were located in the tension side of the samples, the positive response was observed whereas the negative response resulted from positing of carbon fibres in the compression side of the specimens.  Figure 5 shows the type of response observed with BCR1 depending on the placement of carbon fibres. It can be seen that the resistance change with deformation was quite reversible except for the initial 1 or 2 cycles where there was noticeable decrease in resistance. However, in the later cycles, this decrease in resistance became insignificant making the piezoresistive behaviour reversible. This fact can also be observed from the Δ*R*/*R* values listed in Table 5. In the last two cycles, Δ*R*/*R* values were the same in most of the cases. The other braided composites (BCR2 and BCR3) also showed similar positive and negative responses. However, the extent of resistance change with deformation was different for different BCRs due to difference in the composition. The fractional change in resistance in different cycles and the average gauge factors for different BCRs are listed in Table 5. It can be noticed that the highest strain sensitivity or gauge factor was obtained with BCR1 and the strain sensitivity decreased with the increase in carbon fibre percentage in the BCR composition. The influence of carbon fibre content and positioning on the piezoresistive behaviour has been explained in detail in the next section.

### 3.2. Mechanism of Piezoresistivity

**Figure 6:**
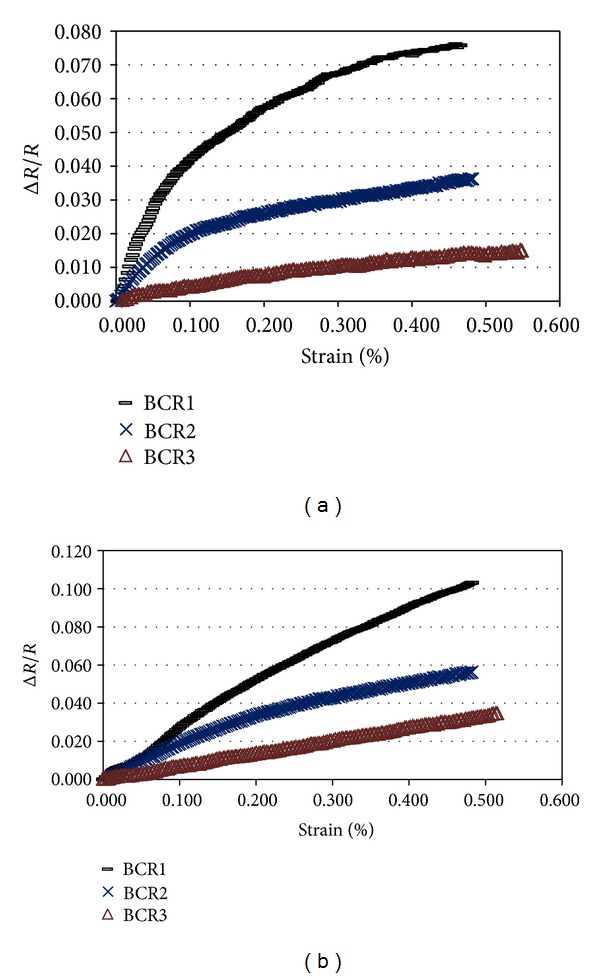
Change of fractional resistance with flexural strain for different BCRs: (a) positive response and (b) negative response.

As reported previously [7], the zero-frequency resistance change of carbon fibre composites may be due to (a) dimensional change as a result of elastic deformation of fibres, (b) change of resistivity resulting from change in interfibre contacts due to strain or change in fibre arrangements, and (d) fibre breakage. Since the composites were subjected to a low strain level in the present study and the piezoresistive behaviour was quite reversible, the effect of dimensional change and fibre breakage was expected to be negligible. The role of interfiber contacts was believed to be the dominating factor for resistance change in the studied braided composites. The change of inter fibre electrical contacts upon flexural loading was probably attributed mainly to two facts such as, first, *(a) separation of carbon fibres*. As, in the flexural loading, different sections of BCR cross-section were subjected to different level of tensile stresses (in the tension side of the specimen) according to their distance from the neutral axis, the fibres located in one section may try to separate from the next one due to shearing forces acting on them. This fact results in the decrease of number of electrical contacts leading to increase in the electrical resistance. The increase in electrical resistance of carbon fibre composites under flexural deformation was also observed in other studies; however, the mechanism was not explained [32]. In the compressive side of the specimens, however, compressive stresses probably lead to fibre bulking resulting in more touching of fibres and decrease in the electrical resistance. The second fact is *(b) fibre alignment*. Upon application of flexural loads, the misaligned carbon fibres in the tension side of the cross-section get oriented along the BCR axis. This fact may aggravate the fibre separation phenomena and lead to further increase in the electrical resistance. However, fibre alignment phenomena is absent in the compressive side of the specimens and, therefore, these misaligned fibres do not have much influence on the resistance change of the compressive side. Another important point that needs attention here is that when the carbon fibres are subjected to uniform tensile stresses throughout the cross-section of BCR, as in case of tensile leading, fibre alignment can help to increase the electrical contact points (as the uniform tensile stresses try to orient the carbon fibres and form a compact bundle of fibres) resulting in decrease in the electrical resistivity, as observed in the previous studies [34]. So, according to this mechanism, the change of electrical contact points is expected to be more with misaligned fibre arrangements due to the possibility of fibre alignment upon deformation. The misaligned arrangement of conductive carbon fibres, therefore, resulted in very good strain sensitivity of the studied BCRs. It can be noticed that the highest piezoresistive behaviour was obtained with BCR1 and the strain sensibility decreases with the increase in the carbon fibre %. In the composites with higher amount of carbon fibres, there will be less change in electrical contacts during deformation due to more touching of fibres leading to a large number of electrical contact points throughout the composites. Previous researchers also found less strain sensitivity with higher percentage of carbon fibres in tensile loading due to the decrease in the “electrical ineffective length” [35] (average length between two adjacent contact points of misaligned carbon fibres) with the increase in carbon fibre percentage [9]. Moreover, with higher amount of carbon fibres, it was not possible to restrain the position of carbon fibres only in one half of the cross-section and therefore the carbon fibres experienced both tensile and compressive stresses. As a result, the overall strain sensitivity decreased due to cancelling effect of positive and negative responses. The trend of fractional resistance change with strain in the first cycle has been presented in Figure 6. It is interesting to note that the curve for BCR1 presents more nonlinearity than the other BCRs. The fractional resistance changes sharply and linearly up to 0.1% strain and then more gradually at higher strains due to saturation in the electrical contacts. This behaviour was also observed in continuous carbon fibre composites where resistance change was mainly attributed to the change in electrical contact points [35]. Another interesting point to note here is that the negative responses seem to be more linear than the positive responses. In case of positive response, as carbon fibres are mainly located in the tension side of the specimen, the change of electrical contact points due to alignment of disoriented fibres under tension is significant in very low strain and as the strain increases, this effect disappears when the fibres become oriented and the effect of fibre separation only plays the main role. Because of this, the change of resistance at low strain level is high and gradually the resistance changes levels off as the strain increases. However, in case of negative response, where the carbon fibres are subjected to compressive forces, the effect of fibre alignment is absent and due to this, resistance changes more uniformly throughout the strain cycle.

### 3.3. Mechanical Performance

The results of tensile testing of BCRs are listed in Table 6. It can be seen that the highest tensile strength and breaking stain were obtained in case of BCR1, that is, composites with 77% of E-glass and 23% carbon core fibres. As the glass fibres present in these composites are at higher quantity, they can bear the redistributed loads even after breakage of carbon fibres. Moreover, the loads can also be transferred back to the broken carbon fibres and partially sustained by means of a mechanism called positive hybrid effect [11] leading to higher breaking strength and extension. However, the breaking extension of BCR1 is still lower than the carbon fibres (1.8%) indicating insufficient use of carbon fibres in the braided composites. This is due to the misalignment of core fibres under the influence of braiding process. Although pretensioning of core fibres helps to maintain their alignment and improves the mechanical performance of BCRs [27], this has been avoided in the present study since misalignment of core fibres was found helpful for strain sensitivity. Due to the less amount of carbon fibres, BCR1, however, showed lower elastic modulus as compared to BCR3 comprised of 100% carbon fibres. The lowest tensile properties were obtained with BCR2 which was made of 53% E-glass and 47% carbon fibres. As the glass fibres that were present in these braided composites had almost equal quantity to that of the carbon fibres, it was not possible for them to bear the load sustained by the composites after the breakage of carbon fibres. This resulted in catastrophic failure and poor mechanical properties. Besides composition, the mechanical performance of BCRs can be further tailored by changing the grade or type of glass and carbon fibres used.

### 3.4. Optimum Composition

Therefore, the best piezoresistive behaviour was obtained with BCR1 which was also found fairly well in terms of mechanical properties. The gauge factor obtained with this braided composite was either 23.4 or 14.9 (depending on positive or negative response), which is much higher than the other carbon fibre/glass fibre hybrid systems reported previously [5, 9, 32] and also comparable to continuous or short carbon fibres composites [34, 36]. The used braiding process was able to introduce a certain degree of misalignments into the core carbon fibres and also position them in only one-half of the cross-section in order to subject them to either tensile or compressive stresses under flexural deformation. As a result, BCR1 proved to be very effective in sensing very low flexural strain in continuous manner. Although the BCRs containing lower amount of carbon fibres than the one reported in this paper were found better in terms of strain sensitivity, they were not suitable for reinforcement of concrete due to lower mechanical properties as compared to steel rebars [28]. Since these BCRs were primarily developed for concrete reinforcement, both reinforcing and sensing capabilities were taken into account and BCR1 has been considered as the optimum composition. However, for other applications, which allow to use BCRs with lower mechanical properties, compositions with lower amount of carbon fibres can be used to obtain better strain sensitivity. Studies are also underway to improve the strain sensitivity of BCR without reducing the amount of carbon fibres by dispersing short carbon fibres in the matrix [31].

### 3.5. Piezoresistive Behaviour of BCR Reinforced Mortar

**Figure 7:**
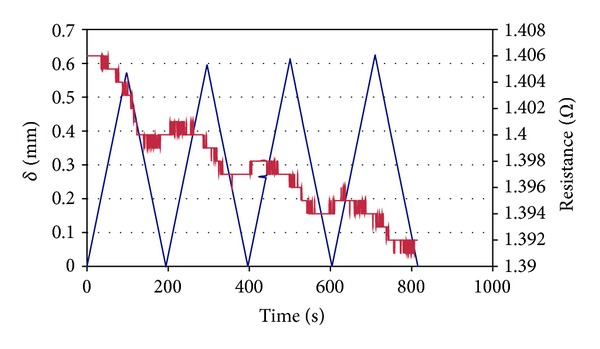
Piezoresistive behaviour of BCR reinforced mortar under cyclic flexural loading.

The test results of piezoresistivity of BCR1 reinforced mortar samples are provided in Table 7 and presented in Figure 7. It can be observed that the resistance decreases in the loading cycles and remains constant in the unloading cycles. Therefore, resistance change is not reversible and continuous loading and unloading cycles lead to the decrease in resistance of BCR reinforced mortar beams. This indicates the permanent changes in the electrical contact points in each cycle, probably due to permanent change in the structure of BCRs. This permanent change in BCR structure was possible due to the comparatively higher flexural strain (~1%) used in the piezoresistive characterization of BCR reinforced mortar beams. In this case, the resistance decreased as the BCR was placed in the tension side of concrete samples in a small section where tensile stresses were approximately uniform. This led to alignment of carbon fibres and increase in the electrical contact points, as discussed in “Mechanism of Piezoresistivity” section. Nevertheless, the decrease in electrical resistance, as observed up to this strain level, suggests only minor structural changes and absence of breakage or major structural damage which can increase the electrical resistance significantly. Therefore, a slight increase in resistance can be considered as an alarm signal for possible structural damage in order to carry out the maintenance work on time. It can be also noticed that the BCR reinforced mortar beams showed much lower sensitivity to the change in flexural strain as compared to BCRs. Further study is underway to understand the piezoresistive behaviour of BCR reinforced mortar by estimating the actual stresses to which BCRs are subjected within the mortar beams. Also, studies are being carried out to investigate the piezoresistive behaviour of BCR reinforced mortar by placing the BCRs at different cross-sections of the mortar beams and also at different strain levels. However, it can be concluded from the present results that BCR reinforced beams can sense their own deformations and may be helpful to diagnose structural damage. However, further understanding and improvement are extremely necessary to successfully apply these materials in practical situations.

## 4. Conclusions

This paper reported the potential of core-reinforced hybrid carbon fibre/glass fibre braided rods for continuous monitoring of very low flexural strain. The effect of carbon fibre/glass fibre weight ratio on both strain sensitivity and tensile properties was investigated. Also, the influence of carbon fibre positioning on strain sensitivity was studied. It was observed that the studied braided composites with lowest amount of carbon fibres (23%) led to best strain sensitivity and good mechanical properties. The change of resistance in the braided composites was mainly attributed to the resistivity change due to strain-dependent change in the electrical contacts either due to fibre separation or fibre alignment or both. The braided composites showed both positive and negative responses under flexural strain depending on the placement of carbon fibres in the cross-section. The much higher gauge factors obtained with the best composition at low strain level (23.4 for positive response and 14.9 for negative response at strain up to 0.55%) as compared to previously reported hybrid composites were due to the misaligned arrangement of carbon fibres caused by the braiding process. Mortar beams reinforced with the BCRs having optimum composition were also found to sense their flexural deformation. However, the piezoresistive behaviour of reinforced beams was irreversible and may be useful to detect the structural damage. Further studies are going on to properly understand the piezoresistive behaviour of BCR reinforced mortar in order to successfully apply them in real situations.

## Figures and Tables

**Table 1:** Properties of core reinforcement fibres.

Fibre type	Manufacturer	Elastic modulus (GPa)	Tensile strength (GPa)	Strain at break (%)
Carbon	Toho Tenax	240	4.3	1.8
E-glass	Saint-Gobain Vetrotex	73.5	3.5	4.8

**Table 2:** Composition of different braided composites.

Codes	Fibre weight fraction	Diameter (mm)	Core fibre type	Core composition (wt%)
BCR1	0.35	5.27	E-glass/carbon	77/23
BCR2	0.32	5.75	E-glass/carbon	53/47
BCR3	0.33	6.40	carbon	100

**Table 3:** Testing parameters for piezoresistive characterization of BCR.

Parameters	Values
No. of cycles	4
Span length (mm)	60
Sample length (mm)	138
Displacement limit (mm)	0.55
Crosshead speed (mm/min)	0.3

**Table 4:** Testing parameters for piezoresistive characterization of BCR reinforced mortar.

Parameters	Values
No. of cycles	4
Span length (mm)	90
Sample length (mm)	105
Force limit (kN)	2.5
Crosshead speed (mm/min)	0.3

**Table 5:** Fractional resistance change and average gauge factor of BCRs.

Cycle no.		1		2		3		4		Average GF
BCR type	Response	*ε* (∗10^−2^)	Δ*R*/*R*	*ε* (∗10^−2^)	Δ*R*/*R*	*ε* (∗10^−2^)	Δ*R*/*R*	*ε* (∗10^−2^)	Δ*R*/*R*	
BCR1	Positive	0.48	0.10	0.48	0.11	0.48	0.12	0.48	0.12	23.4
	Negative	0.47	0.08	0.47	0.07	0.47	0.07	0.47	0.06	14.9
BCR2	Positive	0.48	0.04	0.48	0.02	0.48	0.01	0.48	0.01	4.2
	Negative	0.48	0.06	0.48	0.07	0.48	0.07	0.48	0.07	14.1
BCR3	Positive	0.55	0.02	0.55	0.01	0.55	0.01	0.55	0.01	2.3
	Negative	0.52	0.04	0.52	0.04	0.52	0.05	0.52	0.05	8.6

**Table 6:** Tensile properties of BCRs.

BCR type	Modulus of elasticity (GPa)	Tensile strength (MPa)	Extension at failure (%)
BCR1	78.5	766.7	1.4
BCR2	74.5	740.4	1.2
BCR3	96.3	747.8	1.2

**Table 7:** Results of piezoresistive characterization of BCR reinforced mortar.

Parameters	Cycles			
	1	2	3	4
*t* (s)	97	296	500	710
*δ* (mm)	0.571	0.595	0.612	0.624
*R* (Ω)	1.403	1.399	1.397	1.394
Δ*R*/*R*	0.002	0.005	0.006	0.009
*ε* (∗10^−2^)	1.057	1.102	1.133	1.156
